# A Hybrid Approach for Modeling Type 2 Diabetes Mellitus Progression

**DOI:** 10.3389/fgene.2019.01076

**Published:** 2020-01-07

**Authors:** Sajida Perveen, Muhammad Shahbaz, Muhammad Sajjad Ansari, Karim Keshavjee, Aziz Guergachi

**Affiliations:** ^1^ Department of Computer Science & Engineering, University of Engineering & Technology, Lahore, Pakistan; ^2^ Research Lab for Advanced System Modelling, Ryerson University, Toronto, ON, Canada; ^3^ Division of Science and Technology, University of Education, Lahore, Pakistan; ^4^ Institute for Health Policy, Management and Evaluation, University of Toronto, Toronto, ON, Canada; ^5^ Ted Rogers School of Information Technology Management, Ryerson University, Toronto, ON, Canada; ^6^ Department of Mathematics & Statistics, York University, Toronto, ON, Canada

**Keywords:** type 2 diabetes mellitus, machine learning, hidden Markov model, prognostic modelling, risk prediction, risk scoring

## Abstract

Type 2 Diabetes Mellitus (T2DM) is a chronic, progressive metabolic disorder characterized by hyperglycemia resulting from abnormalities in insulin secretion, insulin action, or both. It is associated with an increased risk of developing vascular complication of micro as well as macro nature. Because of its inconspicuous and heterogeneous character, the management of T2DM is very complex. Modeling physiological processes over time demonstrating the patient’s evolving health condition is imperative to comprehending the patient’s current status of health, projecting its likely dynamics and assessing the requisite care and treatment measures in future. Hidden Markov Model (HMM) is an effective approach for such prognostic modeling. However, the nature of the clinical setting, together with the format of the Electronic Medical Records (EMRs) data, in particular the sparse and irregularly sampled clinical data which is well understood to present significant challenges, has confounded standard HMM. In the present study, we proposed an approximation technique based on Newton’s Divided Difference Method (NDDM) as a component with HMM to determine the risk of developing diabetes in an individual over different time horizons using irregular and sparsely sampled EMRs data. The proposed method is capable of exploiting available sequences of clinical measurements obtained from a longitudinal sample of patients for effective imputation and improved prediction performance. Furthermore, results demonstrated that the discrimination capability of our proposed method, in prognosticating diabetes risk, is superior to the standard HMM.

## Introduction

Diabetes mellitus is a metabolic disorder of multiple etiologies (Alberti and Zimmet, 1998). It can lead to progressive development of multidimensional complications as to vascular system of human body (Einarson et al., 2018). Complications of micro-vascular endpoints may include retinopathy, nephropathy and neuropathy, while the ones related to macro-vascular endpoints may include macro-vascular endpoints may include stroke, peripheral vascular disease and ischemic cardio vascular disease (McEwen and Herman, 2017; Zou et al., 2018). Diabetes mellitus is found to be potentially an independent contributing factor for premature mortality and reduced life expectancy (Atlas, 2015). There is significant evidence that the prevalence of diabetes mellitus is rising shockingly at a faster pace affecting middle-aged adult population disproportionately (Emerging Risk Factors Collaboration, 2010). Globally, about 382 million people were diagnosed with diabetes in 2013—bringing 6.6% of the world’s population under this disease (Perveen et al., 2016). Studies indicate it is likely to escalate by 51% by 2030 (Wild et al., 2004).

Diabetes accounts for a considerable proportion of health-care resources worldwide (Liebl et al., 2000; Perveen et al., 2018a). Even though a century after the invention of insulin, diabetes still calls for significant therapeutic measures. Degenerative complications (like renal failure and cardio vascular disease) in a substantial fraction of diabetic patients are the reasons behind it to some extent (Pambianco et al., 2006; Gregg et al., 2014). Health-care cost for diabetic patients is anticipated to be about $490 billion for 2030, which accounts for 11.6% of global health spending (Perveen et al., 2016).

Several pathogenic processes are believed to play long-winded role in the development process of diabetes (Perveen et al., 2018a). Diabetes does not manifest noticeable symptoms at the initial stage of its development (Ramachandran, 2014). Rather, it demonstrates a highly covert nature of symptoms particularly at the early stage of disease until it substantially developed and evident symptoms transpire (American Diabetes Association, 2014). Due to this asymptomatic intricacy of diabetes, the identification a-priori of pre-diabetic individuals remains quite challenging.

In 2002, Diabetes Prevention Program (DPP) demonstrated that lifestyle intervention directed at exercise and reducing weight was more effective to reduce or delay the risk of developing Type 2 Diabetes Mellitus (T2DM) than the *treatment* with Metformin (Knowler et al., 2002; Lindstrom et al., 2003; Li et al., 2008). Several meta-analysis and clinical trial also suggests that early interventions can delay or altogether counteract the developing mechanism of diabetes mellitus (Li et al., 2008). However, the constraints and cost of these interventions for individuals are primary the arguments against their provision. Furthermore, the interventions can be cost effective only when appropriate target population is used i.e. the one which has a high likelihood of developing diabetes at the baseline (Diabetes Prevention Program Research Group, 2003).

Within this context, the focus of disease management needs to be changed as follows; from hazard to vulnerability reduction; from reactive to proactive; from response management to risk management. However, these changes require novel technological solutions with an emphasis on management of early stages of the disease.

There are various well know diabetes risk prediction model, including FDRSM (Wilson et al., 2007), ARIC (Kahn et al., 2009), San-Antonio (Stern et al., 2002), AUSDRISK (Chen et al., 2010) and FINDRISC (Lindström and Tuomilehto, 2003) that provide the opportunity to estimate the risk of developing diabetes. However, the selection of appropriate risk scoring model is a cumbersome and challenging process (Mashayekhi et al., 2015; Perveen et al., 2016). In general these scoring models are based on prospective studies (like Framingham heart study (Wilson et al., 2007) that prove to be very expensive and also time consuming. Furthermore, these risk scoring models also inherit bias due to differential loss to follow up along with a progressive time taking and costly screening procedure that again makes the intervention measures ineffective and impractical.

Machine Learning (ML) techniques, over the last few years, have been seen to exhibit an increased relevance to a variety of objectives, including risk assessment (Perveen et al., 2018b). This rich knowledge may be useful for some decisive steps to characterize disease risk and progression. ML techniques seem to be an appealing option for the prevention of T2DM. In this context, Electronic Medical Records (EMRs) create a promising horizon for establishing rich and complex physiological models (Gunter and Terry, 2005; Liu et al., 2015). Hence, it is a driving factor for the adoption of state of art data-driven techniques, bringing together the opportunities to automate health-care related tasks (Birkhead et al., 2015).

Hidden Markov Model (HMM) has been extended to deal with the sequential data (Lai et al., 2016). It is particularly an effective approach to predict the future risk of a disease in an individual using sequences of clinical measurements obtained from longitudinal samples of patients (El Nahas et al., 2012; Srikanth, 2015). While classical HMM is used for disease progression modeling, in general, it is not suitable because it assumes that measurement data is collected regularly at discrete time intervals (Liu et al., 2015). However, in reality patient visits are often irregular in time, as a consequence of scheduling issues, selectively miss some pre-scheduled visits or be assessed at self-selected points in time and changes in symptomatology (i.e. patients may visit more often when unwell or vice versa). Consequently, yield electronic medical records with observations sequence irregularly or sparsely sampled and grossly violate the model assumption. Furthermore, these effects also make learning and inference problems more complicated.

In order to resolve the above-mentioned problem and to provide a prompt and comprehensive analysis of EMRs data in the present study, we propose to use HMM with a formulation approach based on Newton’s Divided Difference Method (NDDM) to develop a simple and robust tool to investigate the future diabetes incidents by learning dynamic interactions from longitudinal data. The early identification of pre-diabetic individuals, even when they are in a normoglycemic state, provides further reason for targeting interventions in those, most likely to benefit. Furthermore, the utility of the proposed formulation approach in conjunction with standard HMM has not been explored to address the problem of sparse and irregularly sampled EMRs data which is an unavoidable issue in almost every health-care dataset.

## Materials and Methods

### Study Design, Participants and Data Collection

This prospective study primarily focuses on EMRs data obtained from the Canadian Primary Care Sentinel Surveillance Network (CPCSSN) (http://cpcssn.ca/). This prospective dataset contains 812,007 records of 172,168 unique individuals, those were enrolled in CPCSSN between the years 2003 and 2015.

Temporal feature vectors of clinical measurements for each patient was generated based on the patient’s extracted EMRs data from the observation window. Each feature vector representation includes information related to BP (Blood Pressure), sex, Body Mass Index (BMI), Fasting Blood Glucose (FBG) levels, age, High Density Lipoprotein (HDL), Light Density Lipoprotein (LDL), Glycated Hemoglobin (HbA(1c), total cholesterol and Triglycerides (TG). All patients were assigned a unique ID to track the health status during the follow-up period. In this article we intended to explore the potential of EMRs data to assess T2DM risk, thus we did not add any other outside covariate (i.e. physical activities. To capture a representative cohort, all individuals who have at least 5 visits with 1 year time interval gap till the end of 8 years of follow-up and have information for all the attributes included in this proposed study, as mentioned in **Table 2**, were eligible for inclusion. Approximately 170,250 patients out of 172,168 do not meet minimum inclusion criteria and excluded from the research sample. Thus, this prospective study resulted in a total of 1981 participants for final dataset. In terms of patient demographics, the average age was 40 years, range between 18 to 83 years.

Generally, early risk identification of T2DM in populations is appropriate when it will be an organized continuous process rather than a single time and isolated effort. Furthermore, there should be a reasonable balance in the costs of case identification and treatment in relation to healthcare cost as a whole. Therefore, using these sequences of clinical measurements extracted from CPCSSN a set of experiments was completed to measure how well the proposed data-driven and multivariate predictive model performs in evaluating the ongoing risk of T2DM over varied length of prediction windows.

The primary outcome of interest is to prognosticate risk of developing diabetes in an individual over a series of 8 time horizons: 1 year to 8 years. Such modeling confers an epistemic and instrumental value that manifests in the ability to take intervention measures on time and/or provide individualized treatments based on disease risk (Yoon et al., 2016). By having follow-up through 2015, we ensured that all individuals had at least 5 years of follow-up regardless of disease status.

### Proposed Method

Considering the objective of the proposed research and the above mentioned challenges the proposed method consists of two main components. (1) Handling sparse and irregularly sampled time series EMRs data and (2) the development of prognostic prediction model based on HMM using relevant risk factors for prognostic prediction of diabetes risk over different time horizons.

#### Handling Sparse and Irregularly Sampled EMRs Data

Modeling the clinical condition of a particular patient using evidential physiological data is a ubiquitous problem that arises in many healthcare settings (Liu et al., 2015; Schulam and Saria, 2015; Yoon et al., 2016; Hoiles and Van Der Schaar, 2016). In this context, EMRs data is one of the fundamental resources to derive medical insights and/or support medical practice However, management and processing of such data is challenging due to various factors that are inherent in the data itself.

In particular, the dynamic range of the time scale in EMRs is one of the bothersome characteristics of EMRs data and potentially the contributing factor for sparse and irregularly sampled clinical data. Irregularly sampled (or non-uniformly sampled) time series are characterized by variable time intervals between successive observations (Li and Marlin, 2016). When the intervals between successive observations are long, the time series are said to be sparsely sampled. Irregularity is caused by the fact that patients will only have EMRs data recorded when they visit the hospital.

Consider a longitudinal EMRs data of n independent time series D={S_1_, S_2_,……, S_n_}  recorded at a specific time, for instance, hours, months, or years. Each S_i_ is represented as a list of time points t_i_ = {t_i1_, t_i2_,……,  t_i_| S_i_|}^T^, and a list of corresponding values, y_i_ = {y_i1_, y_i2_,……, y_i_| S_i_ |}^T^. We assume that each time series is defined over a regular time interval [0, T ]. However, for irregularly sampled time series we do not assume that all of the time series are defined on the same collection of time points (i.e.,  t_i_ ≠ t_j_ in general), we do not assume that the intervals between time points are uniform. We also do not assume that the number of observations in different time series is the same (i.e., |S_i_| ≠ |S_j_| in general) and evolves smoothly over time. In other words longitudinal record of each patient is considered as a sparse matrix with features and a time dimension.

CPCSSN data is prone to sparsity and irregularity and tended to violate the HMM based prognostic model assumption to some degree. In addition, sparse and irregularly sampled time series data is itself different from traditional structured data to fit a model. Therefore before developing analytics solution from such data for prognostic prediction of T2DM risk over different time horizons, we propose to use an approximation approach based on NDDM (Kalu, 2009). Which approximates the values for those unknown observations in the longitudinal matrix for each patient by exploring the latent structures on both feature and time dimensions from the information which becomes available from relevant observations in EMRs.

Furthermore, it would be worth exploring whether the proposed method could affect the classification accuracy of the prediction model. However, according to our best knowledge this is the first study that incorporated NDDM to handle the problem of sparse and irregularly sampled EMRs data before developing numerical solution for prognostic prediction of diabetes risk over different time horizons.

NDDM is a standard method used for interpolating polynomial in terms of divided differences. The interpolation problem can be defined as follows: given a set of pairs of numbers (**x**_**0**_, **f**_**0**_), (**x**_**1**_, **x**_**1**_), ………, (**x**_**n**_, **f**_**n**_),  with all **x**_**1**_, **x**_**2**_,……,**x**_**n**_  are different and not necessarily equally spaced, whereas **f**_**i**_  may be the value of some mathematical function **f**(**x**) or empirically obtained in an experiment or observation. The interpolation problem is to find a polynomial **P**_**n**_(**x**) such that **P**_**n**_(**x**)= **f**_**0**_, **P**_**n**_(**x**_**1**_) = **f**_**1**_,…., **P**_**n**_(**x**_**n**_)= **f**_**n**_.

The polynomial P_n_(x) is used to estimate value for all x such that P_n_(x) is approximately f(x) or to get values for x_s_  at which no measurement was taken. This interpolation polynomial can be written in the Newton form as follows (Mathews, 1986):

$${\text{P}}_{\text{n}}(\text{x})=\text{f}[{\text{x}}_{0}]+\text{f}[{\text{x}}_{0},{\text{x}}_{1}](\text{x}-{\text{x}}_{0})+\text{f}[{\text{x}}_{0},{\text{x}}_{1},{\text{x}}_{2}](\text{x}-{\text{x}}_{0})(\text{x}-{\text{x}}_{1})+\text{f}[{\text{x}}_{0},{\text{x}}_{1},{\text{x}}_{2}{\text{x}}_{3}](\text{x}-{\text{x}}_{0})(\text{x}-{\text{x}}_{1})(\text{x}-{\text{x}}_{2})+\mathrm{......}+\text{f}[{\text{x}}_{0},{\text{x}}_{1},{\text{x}}_{2},\mathrm{...}{\text{x}}_{\text{n}}](\text{x}-{\text{x}}_{0})(\text{x}-{\text{x}}_{1})(\text{x}-{\text{x}}_{2})\mathrm{.......}(\text{x}-{\text{x}}_{\text{n}})$$

Where {f[x_0_],  f[x_0_, x_1_] and f[x_0_, x_1_, x_2_]} are finite divided differences and  f[x_0_], f[x_0_, x_1_] and f[x_0_, x_1_, x_2_] are the first, second, and third order finite divided differences, respectively that can be defined as below:

$$\text{f}[{\text{x}}_{0}]=\text{f}\left({\text{x}}_{0}\right)$$

$$\text{f}[{\text{x}}_{0},{\text{x}}_{1}]=\frac{\text{f}\left({\text{x}}_{1}\right)-\text{f}\left({\text{x}}_{0}\right)}{{\text{x}}_{1}-{\text{x}}_{0}}$$

$$\text{f}[{\text{x}}_{0},{\text{ x}}_{1},{\text{ x}}_{2}]\;=\frac{\;\frac{\text{f}\left({\text{x}}_{2}\right)-\text{f}\left({\text{x}}_{1}\right)}{{\text{x}}_{1}-{\text{x}}_{0}}-\frac{\text{f}\left({\text{x}}_{1}\right)-\text{f}\left({\text{x}}_{0}\right)}{{\text{x}}_{1}-{\text{x}}_{0}}}{{\text{ x}}_{2}-{\text{x}}_{0}}$$

Similarly, n^th^ Divided Difference is given by

$$\text{f}[{\text{x}}_{\text{i}},{\text{ x}}_{\text{i}+1},{\text{ x}}_{\text{i}+2},\ldots ,{\text{x}}_{\text{i}+\text{n}}]=\frac{\text{f}[{\text{x}}_{\text{i}},{\text{ x}}_{\text{i}+1},{\text{ x}}_{\text{i}+2},\ldots ,{\text{x}}_{\text{i}+\text{n}}]-\text{f}[{\text{x}}_{\text{i}},{\text{ x}}_{\text{i}+1},{\text{ x}}_{\text{i}+2},\ldots ,{\text{x}}_{\text{i}+\text{n}-1}]}{{\text{ x}}_{\text{i}+\text{n}}-{\text{x}}_{\text{i}}}$$

#### Prognostic Modeling

Once the dataset is prepared by taking the output of the proposed approximation method the next crucial task is potentially contributing risk factors selection. Therefore, to optimally select the potentially contributing factors, Logistic Regression (LR) analysis is performed on the derived dataset that consist of risk factors related to demographics, vitals, diagnoses and laboratory tests results as given in **Table 1**.

**Table 1:** Characteristics of the population in the CPCSSN database.

Predictors	Findings
Demographic (Sex, Age)	
Female, sample size (%)	100,566 (57)
Male age mean ± SD, Yr	48.2 ± 24.1
Female age mean ± SD, Yr	49.5 ± 24.8
Male age mean ± SD, Yr	48.2 ± 24.1
Vital Signs/clinical measures	
Systolic BP, mean ± SD, mm Hg	129.34 ± 17.183
Chronic obstructive pulmonary disease frequency (%)	9,939 (2.4)
Dementia frequency (%)	12,007 (1.8)
Depression frequency (%)	32,672 (10)
Diabetes Mellitus frequency (%)	26,077 (6)
Epilepsy frequency (%)	5,553 (0.8)
Hypertension frequency (%)	61,370 (13)
Osteoarthritis frequency (%)	37,274 (7)
Parkinson’s Disease frequency (%)	1,825 (0.2)
Lab Values	
Fasting blood glucose, mean ± SD, mmol/L	5.54 ± 1.91
TG, mean ± SD, mmol/L	1.523 ± 0.962
LDL, mean ± SD, mmol/L	2.83 ± 0.99
High density lipoprotein, mean ± SD, mmol/L	1.3893 ± 0.416
BMI, mean ± SD, kg/m^2^	37.113 ± 1528.71
HbA(1c), mean ± SD, mmol/L	6.268 ± 0.976
Cholesterol mean ± SD, mmol/L	4.893 ± 1.159

SD, standard deviation; Yr, year; BP, blood pressure; LDL, light density lipoprotein; HbA(1c), glycated hemoglobin; TG, triglycerides; BMI, body mass index; HDL.

* Some patients have more than 1 disease in the database.

Models were trained and evaluated using only risk factors that exhibit significant relationship with T2DM when LR analysis was performed. Models were trained and evaluated using only risk factors that exhibit significant relationship with T2DM when LR analysis was performed. Subsequently, the parameters are drawn from training dataset using Baum-Welch algorithm. However, to fit the predictive model, we used standard GaussianHMM, a variant of classical HMM. It is a finite probability density distribution model that has been widely deployed as temporal latent variable model for modeling dynamic systems (Kenny et al., 1990; Artières et al., 2000). Several variants of the basic hidden markov model have been proposed, with slightly different functionality (Rabiner, 1989). The basic concept was published in a series of classic papers by Baum and Petrie (Baum and Petrie, 1966). As our data retained continuous variables thus, the observation probability assumes the Gaussian distribution. Our model has structural assumptions about the underlying structure of the process and assumed to be composed of the set of hidden states  S = {s_1_, s_2_, s_3_……s_m_}  (corresponding to diabetic or non-diabetic in our case) in the model, initial state distribution, an observational symbol distribution (e.g. Gaussian) of each state and a state transition matrix generally parameterized by a set probabilities used for further analysis, as follows:

$$\begin{array}{l} \text{θ}=(\text{ π}=\underset{\text{Prior probability },}{\underset{︸}{{\text{π}}_{\text{i}}=\left\{{\text{q}}_{1}={\text{s}}_{\text{i}}\right\}}}\text{ A}=\underset{\text{Transition propabilities matrix }}{\underset{︸}{{\text{a}}_{\text{i},\text{j}}=\text{p}({\text{q}}_{\text{t}+1}={\text{s}}_{\text{j}}|{\text{q}}_{\text{t}}={\text{s}}_{\text{i}})}}\\ \text{ B}=\;\underset{\text{Emission probabilities matrix}}{\underset{︸}{{\text{b}}_{\text{i}}\left(\text{K}\right)={\text{ b}}_{\text{i}}\left({\text{O}}_{\text{t}}={\text{V}}_{\text{k}}\right)\;=N\left({\text{V}}_{\text{k}},{µ}_{\text{i}},{\text{ σ}}_{\text{i}}\right)}}), \end{array}$$

where µ_i_ and σ_i_ are the mean and variance of the distribution corresponding to the state s_i_ respectively, and 𝒩 is Gaussian probability density function that can be defined as below:

$$\text{p}\left(\text{x}|µ,\text{ σ}\right)=N\;(\text{x}|µ,\text{ σ})={\frac{1}{\sqrt{2\text{πσ}}}}_{0}\text{exp}\left(\frac{-{\left(\text{x}-\text{µ}\right)}^{2}}{2\text{σ}}\right)$$

Hence, the standard Gaussian HMM is specified by λ = {A, µ, σ, π}.

Hold-out method was used in all modeling iterations to obtain an estimate of how well the model can generalize to an independent dataset. Thus, two subset of sequence of clinical measurements were considered, a training set and testing set. 80% of the data was used for training and the remaining 20% for testing.

Subsequently, for each of the 8 time horizons, Viterbi decoding method from HMM API (Hmmlearrn) was incorporated to train Gaussian HMM and carry out diagnostic and prognostic inferences related to diabetes risk in an individual over different time horizons. This is typically a Maximum a posteriori (MAP) estimation of the most likely sequence of hidden states, produced by the Viterbi algorithm given the observation sequence $\text{O}=\left\{O_{\text{t}}^{\left(\text{l}\right)},\text{ t}=1,2,,\ldots .\text{T},\text{ l}=1,2,3,\;\ldots .\text{L}\right\}\;$and O_t_ *ϵ*R^D^  where T is the length of each sequence and l is the numbers of independent observation sequences, and model λ = {A, µ, σ, π }. For further detail supplementary material is given in Presentation 1_v1.pdf.

After training the predictive model, the second task corresponds to the performance evaluation of Gaussian HMM at each of the 8 time points. Therefore, we estimated the discriminatory ability of each model *via* the Area under the Receiver Operating Characteristic Curve (AROC) computed over hold-out method. IBM SPSS Statistics (version 19) was used to perform statistical analysis in this study. Along with this the experiments used a combination of software tools developed in house and based on open source packages for Python (Version 2.7).

## Results

During 2003 to 2015, 172,168 individuals’ of aged between 18 to 90 received healthcare services at CPCSSN, contributing over 8 million records, of these individuals, 40,317 individuals have diabetes, accounting for 23% of all cases morbidity during the study period. After applying inclusion criteria and approximation method, as mentioned above, to deal with irregularly sampled data the final dataset resulted in a total of 1918 individuals with 15,344 clinical visits recorded over 8-years. Approximately 584 (30.44%) of individuals in our derived research sample were diabetic and among them 40.40% were women. Descriptive statistics of eligible cohort are reported in **Table 2**.

**Table 2:** Descriptive statistics of Diabetic and Non-diabetic population in our derived data sample.

Predictors	Findings		
	Total population	Progressors	Non-progressors
	1,918	584 (23.49)	1,334
Demographic (Gender, Age)			
Male, sample size (%)	114 3(38.96)	280	495
Female, sample size (%)	775 (61.03)	304	839
Overall age mean ± SD,Years	63.19± 11.89	65.312 ± 12.34	58.937 ± 14.315
Vital Signs/clinical measures			
Systolic BP, mean ± SD, mm Hg	128.611 ± 15.86	131.49 ± 16.7	128.496 ± 17.259
Lab Values			
FBS, mmol/L mean ± SD, mmol/L	6.029± 1.51	7.256 ± 2.056	5.214 ± 0.562
Triglycerides, mean ± SD, mmol/L	1.72 ± 1.02	1.777 ± 1.205	1.419 ± 0.837
HDL, sample size, mean ± SD, mmol/L	1.356 ± 0.39	1.249 ± 0.361	1.453 ± 0.423
HbA(1c), mean ± SD, mmol/L	6.268 ± 0.95	6.821 ± 1.049	5.698 ± 0.365
Total Cholesterol mean ± SD, mmol/L	5.409 ± 0.59	4.433 ± 1.224	5.081 ± 1.085
LDL, mean ± SD, mmol/L	2.442± 0.851	2.427 ± 1.011	3.007 ± 0.939
BMI, mean ± SD, kg/m^2^	29.81 ± 6.362	36.163 ± 1197.454	37.984 ± 1697.225
Depression frequency (%)			
YES	373	112	261
NO	1,544	472	1,073
Hypertension positive cases frequency (%)			
YES	1,107	421	686
NO	809	163	646
Unknown	2		2

SD, standard deviation; BP, bnlood pressure; BMI, body mass index; FBS, fasting blood sugar; LDL, light density lipoprotein; HDL, high-density lipoprotein; HbA(1c), glycated hemoglobin.

**Figure 1:**
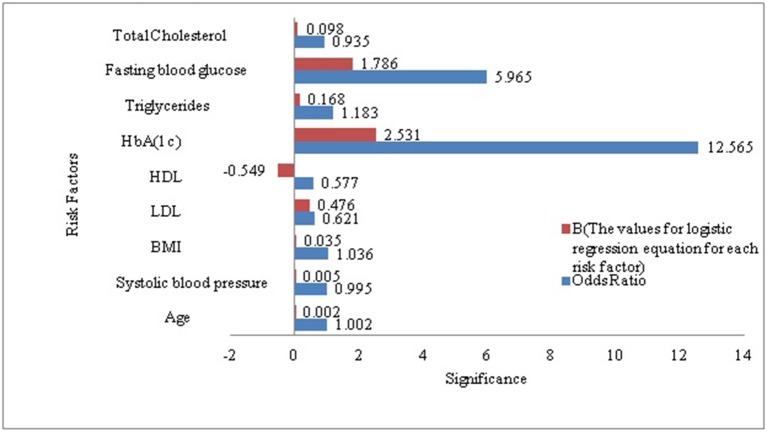
Visualization of the association between diabetes and each of the risk factors included in our study sample.

As a secondary analysis, we also performed a LR analysis to evaluate the significant p-value of each risk factor included in our derived dataset in the context diabetes risk identification. According to the LR analysis except total cholesterol all the risk factors included in our research sample were statistically significant and added value to the model in prognosticating T2DM risk. HbA(1c) (Glycated Hemoglobin) was the most strongly associated with diabetes as compare to other risk factors included in this analysis, it remained the best predictor with odds ratios of (p < 0.0005, OR = 12.565 [95% CI, 10.902 -14.482]). It demonstrated that HbA(1c) solely was the prime risk factor with the ability to prognosticate the diabetes risk. Whereas FBG was ranked at second among the risk factors included in this study for prognosticating the diabetes risk (p < 0.0005, OR = 5.965[95% CI, 5.607 -1.281]). To get a better understanding of what was going on inside the LR and to visualize the relative influence of each predictor for predicting diabetes risk we plotted **Figure 1**. It can also be observed that the B-value (-0.549) for LR equation for predicting the diabetes risk from the HDL is negative although it hold a significant p-value (5.63E-24, 95% CI). It demonstrated that increased level of HDL is associated with a reduced likelihood of diabetes onset (p < 0.0005, OR = .577 [95% CI, 0.480 -0.695]). However the B-values for the remaining risk factors are positive.

**Figure 2:**
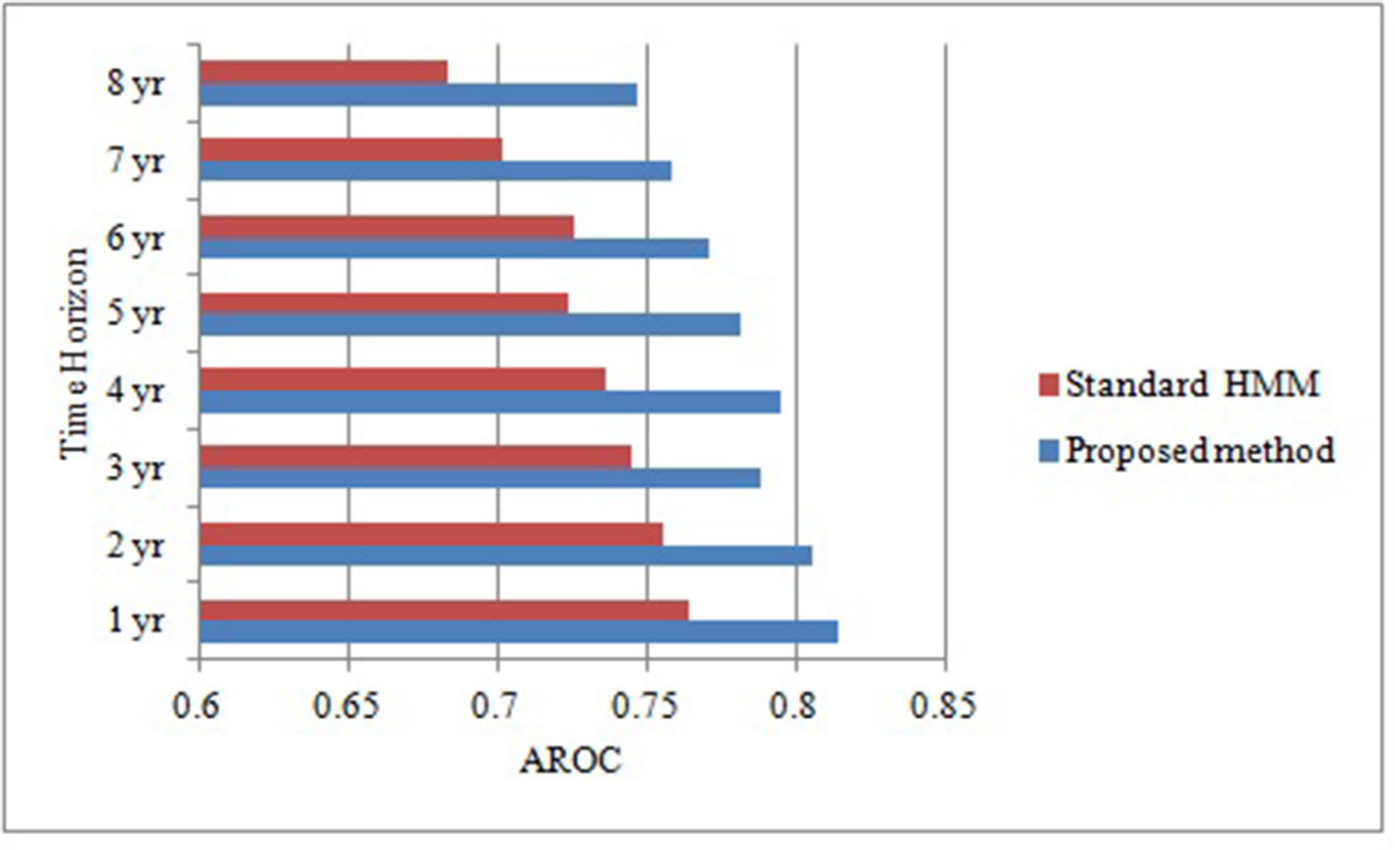
Comparative analysis of area under receiver operating characteristic curve of our proposed method and standard HMM over different time horizons.

As the objective of this proposed research is to prognosticate T2DM risk in an individual over different time horizon in order to make informed choices about future care and treatment with reduced complications and improved outcomes. Therefore, we prognosticate diabetes risk over a series of time horizon using only positively correlated and modifiable risk factors. Although this association provides some general guidance for diabetes but ineffective for individual risk assessment (Arbab-Zadeh and Fuster, 2015). In order to prove our proposed algorithm effectively, we make the contrast experiments. **Figure 2** compares the predictive performance of our proposed method and standard HMM in term of AROCs for developing diabetes risk over different time horizon, using approximated and irregularly sampled data respectively. The AROC of our proposed method on our derived and approximated dataset was 0.81 (p < 0.0005, [95% CI, (0.791-0.847)]) for prediction window of 1-year as compared to AROC 0.764(p < 0.0005, [95% CI, (0.741-0.794)]) with classical HMM without handling sparse and irregularly sampled multivariate time series data. It can be observed that the proposed method demonstrated significant performance over all the baseline models (p < 0.0005) and time horizons. The highest AROC achieved (0.814) belonged to the 1-year model with our proposed approach, as can be observed from **Figure 2**. Furthermore, experimental results also demonstrated that the AROC of our proposed model is consistently superior over all the time horizons as compare to baseline method. However, as expected, performance for both predictive models declines in relation to increasing time horizons. It can be observed that the predictive performance of our proposed model is at or above 0.795 AROC for prediction windows ≤4 years whereas the performance is at 0.771 AROC for a 6-years prediction window. Performance then declines rapidly for prediction window lengths longer than 6 years.

According to the probabilistic prediction of HMM, we estimated 8 years risk of developing diabetes in our study sample, among 3 different risk categories with the cutoff value <3, 3 to 9 and equal to10. We determined that 46% of individuals in our sample had a risk less than 3%; 38% had a risk between 30% to 9% and 16% had a risk equal to10%.

## Discussion

The intensification in diabetes incidence is principal reason of increased diabetes prevalence. Early identification of individuals at high risk is imperative and a practical approach to prevent or delay the onset of diabetes through implementing proactive lifestyle and pharmacological interventions (Diabetes Prevention Program Research Group, 2003). In this context, physiological data contained in electronic medical records (EMRs) is the fundamental source for disease prognostic modeling (Tou et al., 2018). In addition, the rapid evolution in state of the art ML techniques offer a potentially promising means to accelerate discoveries, from EMRs data, which can be readily translated to clinical practice. From the clinicians’ prospective, the development of such risk scoring techniques would allow them to allocate resources and healthcare services optimally and with more confidence (Vogenberg, 2009).

Hidden Markov Models and their variants have been widely deployed for modeling dynamical systems (Rabiner, 1989). These temporal latent variable models have also attained substantial success in various applications (Gruber et al., 2007; Fox et al., 2011). However, the format of the EMRs data together with the nature of the clinical setting poses various significant challenges that confound standard HMM. In particular, the dynamic range of the time scale in EMRs is one of the potentially contributing factor for sparse and irregularly sampled clinical data. Typically the HMM presumes that the training data sample is collected regularly at discrete time intervals. Thus, direct incorporation of EMRs with observations sequence irregularly or sparsely sampled into standard HMM [(e.g. the models in Fox et al. (2011) and Rabiner (1989)] will not suffice for jointly describing the latent states and hence ensuring accurate inferences. This paper presented a new hybrid approach combining approximation technique as a component with Hidden Markov Model (HMM) in order to deal with sparse and irregularly sampled time series data for effectively determining the risk of developing diabetes in an individual over different time horizons. The proposed method is fully modular. It basically incorporated an approximation method based on NDDM to handle multivariate sparse and irregularly sampled data as dynamical systems inputs, followed by the application of HMM based diagnostic predictive model that operates over the regularly spaced time series output provided by the approximation method.

In order to develop the prognostic prediction model we incorporated further two step approach. Therefore, we also incorporated LR analysis in order to identify potentially contributing risk factors of diabetes. In LR analysis we considered 0.05 level of significance, as depicted in **Table 3**.

**Table 3:** Analysis of the association between individual risk factor and diabetes risk in our study sample.

Explanatory variables	OR (95% C.I.)	P Value
Age	1.002 (.999 -1.006)	3.49E-22
Systolic blood pressure	.995 (.993-.997)	7.02E-07
BMI	1.036 (1.030 1 -.042)	1.60E-58
LDL	.621 (.528 -.732)	1.56E-23
HDL	.577 (.480 -.695)	5.63E-24
HbA(1c)	12.565 (10.902 -14.482)	7.94E-143
Triglycerides	1.183 (1.093 -1.281)	5.34E-07
Fasting blood glucose	5.965 (5.607 -1.281)	0.000
Total Cholesterol	.935 (.795-1.098)	0.411
Intercept		3.63E-145

Nagelkerke R2 = 0.546.

Hosmer and Lemeshow Test = 0.360 (Significantly greater than 0.0005).

OR, odds ratio; C.I., confidence interval; sBP, systolic blood pressure; BMI, body mass index; LDL, light density lipoprotein; HDL, high density lipoprotein; HbA(1c), glycated hemoglobin.

**Table 3** shows the results considering a significant level of 0.05. It depicts highly significant association between each risk factor and diabetes expect total cholesterol. This means that all the risk factors added value to the model for diabetes onset prediction excluding total cholesterol. As the total cholesterol exhibited negative association with the T2DM thus we excluded it from the contributing risk factors. Along with this, we also excluded gender from the potentially contributing risk factors. As gender is a non modifiable risk factor; thus, provide not much guidance for diabetes prevention. Wilson et al. (2007) also not recommended gender as a candidate risk factor for the prediction of diabetes onset. On the other hand age is also a non modifiable risk factor but we did not exclude it from contributing risk factors because several existing research conducted by Stern et al. (2002), Lindstrom et al. (2003), Kanaya et al. (2005), Zhou et al. (2013) and Perveen et al. (2016) suggested that age is a potentially significant risk factor for prognostic prediction of diabetes risk. These studies also highlighted that elderly population had higher risk for developing T2DM than those with younger age. In addition, our results also demonstrated that age is a significant influencing factor for diabetes (p < 0.0005, OR = 1.002[95% CI, 0.999 -1.006]) as depicted in **Table 3**.

Nevertheless, HDL is statistically significant risk factor (p < 0.0005, OR = .577 [95% CI, 0.480 -0.695]) but have a negative association with the development of diabetes risk in an individual (Mazzone et al., 2006; Lincoff et al., 2007). According to our analysis the probability of developing diabetes reduced approximately 5% with one unit increase in HDL level.While increased levels of FBS and HbA(1c) in blood is strongly associated with higher risk of developing T2DM. It can also be observed from the concise adds ratios that HbA(1c) is the strongest predictor for prognosticating diabetes risk. Furthermore, Statistical analysis results of our proposed study are also commensurate with the results of the existing research proposed by Mackey et al. (2015), Wang et al. (2016) and Perveen et al. (2018a).

To evaluate the impact of modeling sparse and irregular time series data using approximation technique based on NDDM we conducted a set of experiments on both the original and approximated synthetic dataset. In both cases the baseline model is GausianHMM however the input to one of them is sparse and irregularly sampled time series. The output is a single scalar representing the predicted class along with the probability distribution over a set of class values.

Validation of the accuracy of a prognostic prediction model is often involves plotting observed incidents verses estimated probability to observe visually how close model predictions were to actual predictions (Buijsse et al., 2011). Therefore, we also incorporated AROC to evaluate the discriminatory capability of our proposed model in identify 8-years risk of diabetes.

In the results, the reported AROC for the proposed method on our derived` dataset was 0.81 (p < 0.0005, [95% CI, (0.791-0.847)]) for prediction window of 1-year, showing a high discriminative capability as compared to standard HMM with AROC 0.764 (p < 0.0005, [95% CI, (0.741-0.794)]). It can also be concluded that dealing with sparse and irregularly sampled multivariate time series data can yield relatively better performance. Furthermore, experimental results also demonstrated that the AROC of our proposed model is consistently superior over all the time horizons as compare to standard HMM learned, indicating that the model learned using approximation approach based on NDDM have the potential to discriminate persons who will have the diabetes from those who did not with considerably high performance.

In addition to identifying diabetes risk a-prior, this is the first study that proposed an approximation technique based on NDDM to deal with the sparse and irregularly sampled EMR data before developing prognostic model. Furthermore, the proposed method has the ability to effectively estimate the future risk of T2DM with reduced healthcare expenditures.

The total estimated direct and indirect healthcare expenditures for diabetic patients were $13,700 per year, whereas about $7,900 of this amount was particularly attributed to diabetes (American Diabetes Association, 2013). It is also estimated that diabetic patients have healthcare expenditures, on average, 2.3 times higher than what expenditures would be in the absence of diabetes. As the predictive performance of our proposed method was comparatively good, therefore, we also estimated the 8-years risk of developing T2DM among 1458 non-diabetic individuals for whom data was available in 2015. According to the probabilistic results of our proposed method over the baseline data set it can be observed that approximately 15.8%(231) individuals have significant risk of developing T2DM in the next 8-years interval ranging from 2015 to 2022. Given the newly identified individuals with increased risk of developing T2DM, we can save a considerable fraction of individuals from our baseline data set if healthcare providers promptly manage those vulnerable.

There are some remarkable advances and benefits of the proposed research. In this study we performed prognostic prediction of diabetes risk over a set of time horizons using EMRs data collected for secondary purposes not for research. Therefore it is a time and cost effective approach. According to LR analysis it can also be concluded that HbA(1c) is the strongest predictor for prognosticating diabetes risk and has the ability to solely predict it. As fasting is not prerequisite for The HbA(1c) test and can be conducted at any time of the day. Therefore, it can easily be used in community screening programs or an opportunistic screening in outpatient visits. Furthermore, in our derived dataset each laboratory test (i.e. FBG, LDL, HbA1c, HDL and triglycerides) was measured and recorded for each time horizon in CPCSSN. However, a limitation of the study is that the proposed model was only validated internally caution is required when applied over other populations in order to minimize the bias in generalization.

## Conclusion

In summary, our results demonstrated that the proposed method has the capability to deal with sparse and irregularly sampled data for leveraging EMRs to learn underlying hidden state with the objective to provide insights into the disease process over a series of time horizon. Furthermore, the proposed method conceal the inherent temporal dependencies exist in the temporal data, required for decisive step to characterize disease risk in an individual with significantly improved predictive performance as compare to standard HMM. Therefore, this is an encouraging step forward for active identification of high risk individuals as a means to propel healthcare toward an innovative preventive orientation for diabetes. In future further research is warranted for the cost effective analysis of the proposed study. This can be extended to prognosticate the future risk of other type of ailments particularly chronic diseases.

## Data Availability Statement

The datasets generated for this study are available on request to the corresponding author.

## Ethics Statement

The studies involving human participants were reviewed and approved by Ryerson University research ethics board, Ryerson University, Toronto, Ontario, Canada. The patients/participants provided their written informed consent to participate in this study.

## Author Contributions

All authors contributed equally to the conception, design, and development of the research. SP investigated/predicted the risk of developing type 2 diabetes in an individual using EMRs data. MS provided the technical guidance for conducting the research and analysis of the data. KK critically revised the paper draft for the soundness of the research from the medical viewpoint. AG critically revised the paper draft for the soundness of the research from the machine learning viewpoint. All authors reviewed the manuscript before its submission. MA also provided the technical guidance for conducting the research.

## Conflict of Interest

The authors declare that the research was conducted in the absence of any commercial or financial relationships that could be construed as a potential conflict of interest.
