# Supplementary material for: A Hybrid Approach for Modeling Type 2 Diabetes Mellitus Progression
Source: Front Genet. 2020 Jan 7;10:1076. doi: 10.3389/fgene.2019.01076 (PMC6958689; doi:10.3389/fgene.2019.01076)
Supplement: Supplementary file 1 [file Presentation_1.pdf]

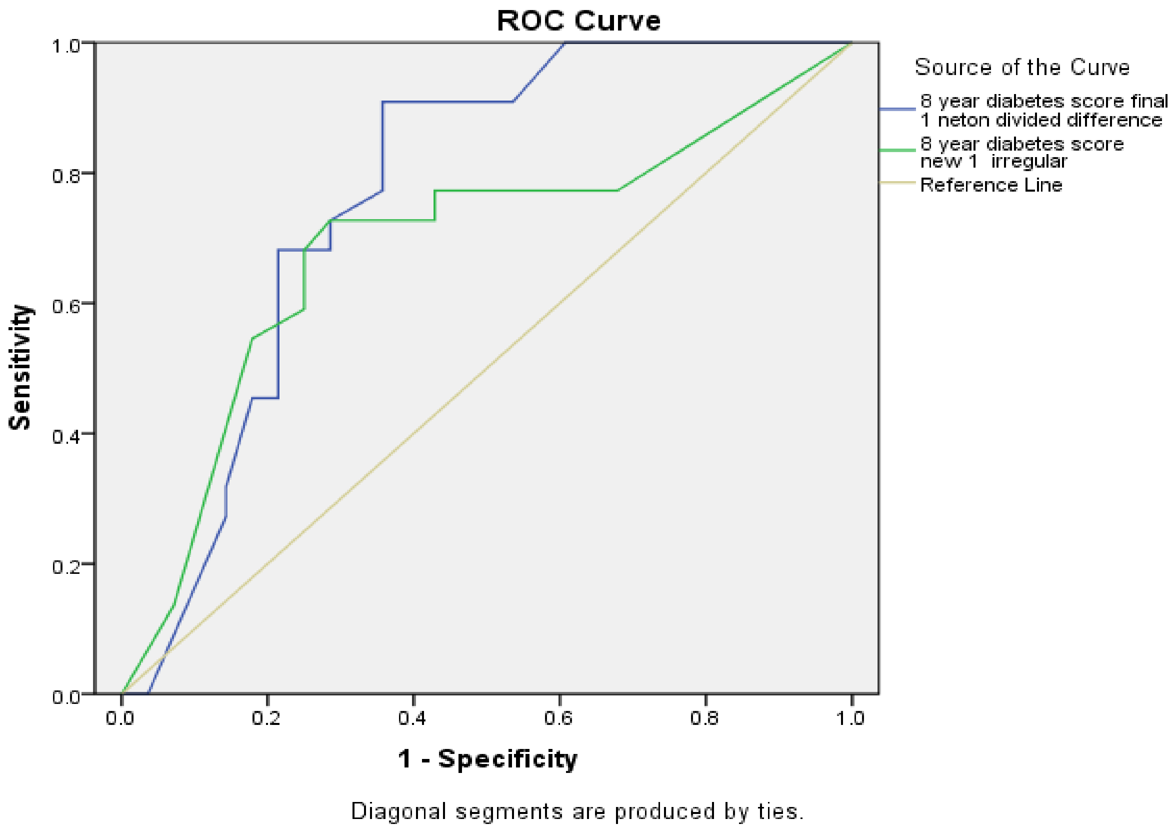

Figure 2: Area under Receiver Operating Characteristic Curve of our proposed method and standard HMM to identify 8 years' risk on approximated and irregularly sampled dataset.

Table 1: Summary of Area Under Receiver Operating Characteristic Curve (AROC) in our derived research datasets.

	AROC	Std. Error [a]	Asymptotic Sig.[b]	Asymptotic 95% Confidence Interval	
				Lower Bound	Upper Bound
Proposed Method/ Performance on approximated data using Newton's divided difference method	0.747	0.068	0 .001	0.637	0.903
Over the derived irregularly sampled dataset	0.653	0.080	0.023	0.533	0.846

The test result variable(s): cal has at least one tie between the positive actual state group and the negative actual state group. Statistics may be biased.

a. Under the non parametric assumption
b. Null hypothesis: true area =0.5

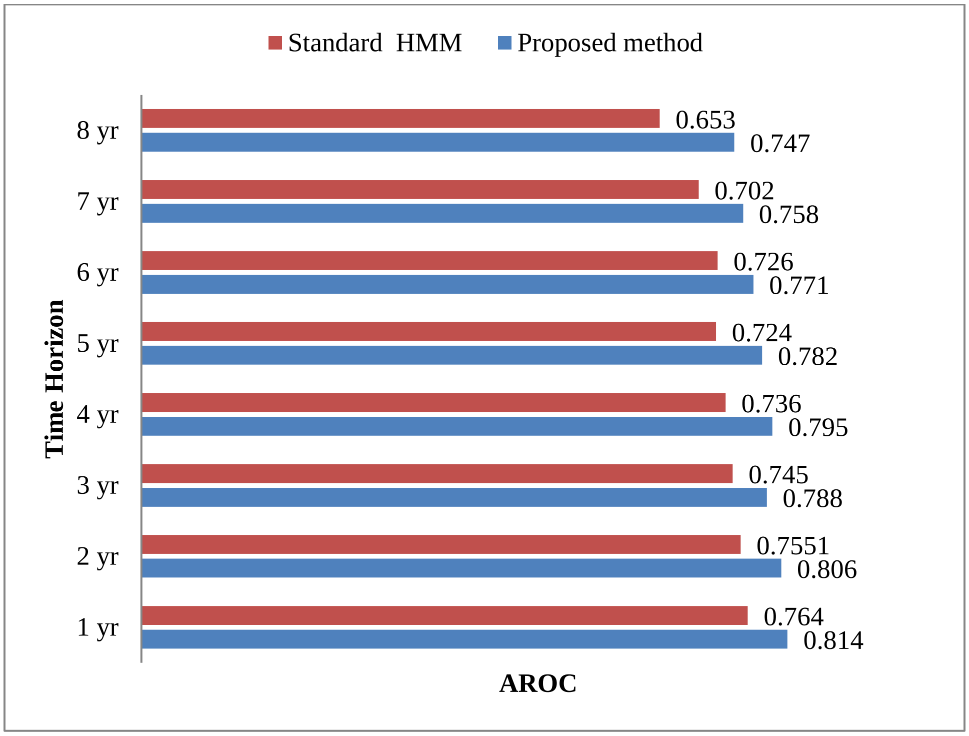

Figure 1: Comparative analysis of Area under Receiver Operating Characteristic Curve of our proposed method and standard HMM over different time horizons.

In the proposed study we have calculated risk of developing diabetes in an individual over 8 different time horizons. Hence, there is 8*2 AROC figures to represent the results. Therefore the ideal method was to represent it in more summarized manner for this purpose we incorporated bar graph with AROC values . However, we also provide only 8 years' risk on both datasets as depicted in Figure 2.

		TP Rate	FP Rate	Precision	Recall	F-Measure	MCC
Proposed Method/ Performance on approximated data using Newton's divided difference method							
	Non-diabetic	0.705	0.184	0.802	0.705	0.75	0.573
	Diabetic	0.761	0.156	0.723	0.816	0.767	0.573
Over the derived irregularly sampled dataset							
	Non-diabetic	0.517	0.312	0.484	0.597	0.528	0.180
	Diabetic	0.596	0.274	0.567	0.0.586	0.581	0.180
